# Successful treatment of a 14-year-old patient with intestinal malrotation with laparoscopic Ladd procedure: case report and literature review

**DOI:** 10.1186/1749-7922-8-19

**Published:** 2013-05-17

**Authors:** Yuka Nakajima, Hiroyuki Sakata, Tomohiro Yamaguchi, Norichika Yoshie, Taihei Yamada, Takaaki Osako, Mariko Terashima, Naomi Mambo, Ryuta Saka, Satoko Nose, Takashi Sasaki, Hiroomi Okuyama, Atsunori Nakao, Joji Kotani

**Affiliations:** 1 Department of Emergency, Disaster and Critical Care Medicine, Hyogo College of Medicine, 1-1 Mukogawa, Nishinomiya, Hyogo 663 8501, Japan; 2 Department of Pediatric Surgery, Hyogo College of Medicine, Nishinomiya, Hyogo, Japan

**Keywords:** Malrotation, Laparoscopic surgery, Ladd procedure, Acute abdomen, Teenager

## Abstract

Midgut malrotation is an anomaly of intestinal rotation that occurs during fetal development and usually presents in the neonatal period. We present a rare case of malrotation in a 14-year-old patient who presented with cramping, generalized right abdominal pain, and vomiting for a duration of one day. A computed tomography abdominal scan and upper gastrointestinal contrast studies showed malrotation of the small bowel without volvulus. Laparoscopy revealed typical Ladd’s bands and a distended flabby third and fourth duodenal portion extrinsically obstructing the misplaced duodeno-jejunal junction. The Ladd procedure, including widening of the mesenteric base and appendectomy, was performed. Symptoms completely resolved in a half-year follow up period. Patients with midgut malrotation may present with vague abdominal pain, intestinal obstruction, or intestinal ischemia. The laparoscopic Ladd procedure is feasible and safe, and it appears to be as effective as the standard open Ladd procedure in the diagnosis and treatment of teenage or adult patients with intestinal malrotation.

## Introduction

Midgut malrotation is a congenital anomaly of intestinal rotation presenting mainly in childhood, usually within the first month of life. Midgut malrotation refers to a failure in the counter-clockwise rotation of the midgut, which results in the misplacement of the duodeno-jejunal junction to the right midline, comprising non-rotation and incomplete rotation of the superior mesenteric artery. Malrotation is typically diagnosed in the first few months of life, and 90% of cases are diagnosed during the first year. However, older children and adolescents are likely to present with recurrent abdominal pain, intermittent obstructive symptoms, or failure to thrive due to intestinal obstruction or intestinal ischemia [1-4].

We present the case of a symptomatic 14-year-old patient complaining of abdominal pain found to have intestinal malrotation that was successfully treated with a laparoscopic Ladd procedure. In adults or older children, the diagnosis is mostly incidental, based on investigation carried out for unrelated symptoms. Indeed, most adult patients are asymptomatic and incidentally, malrotation is often discovered later in life during surgery for other conditions. We diagnosed congenital intestinal malrotation, which rarely occurs in adults or older children, by using several modalities such as barium studies, computed tomography, and laparoscopy. We describe the clinical and radiological data of this patient followed by a brief review of the literature. This case report serves to demonstrate the benefits of laparoscopic surgery for malrotation. Also, the present case reminds us that intestinal malrotation should be considered in the differential diagnosis of a wide variety of symptoms and should be treated promptly once the diagnosis has been confirmed.

## Presentation of case

A 14-year-old man presented to our emergency center with cramping and generalized abdominal pain. His abdominal pain began the previous night shortly after eating and recurred intermittently. Multiple presentations with similar symptoms during his teenage years had failed to identify the cause of his pain. He had no history of previous abdominal surgeries. He was on no medication at the time and denied alcohol or tobacco use. The patient also vomited on the day of presentation with vomitus containing biliary contents. On physical examination, the patient’s vital signs were: pulse, 67 beats/minute; blood pressure, 121/61 mmHg; body temperature, 36.9°C; and respiration rate, 15 breaths/minute. He was well-nourished and alert without cyanosis. His abdomen was not distended, but his bowel sounds were weak. He exhibited no peritoneal signs; however mild diffuse tenderness to deep palpation was noted. His white blood cell count was 10160 /mm^3^. Serum biochemistry and liver function test results were within normal limits, except a C-reactive protein level of 4.2 mg/dl.

**Figure 1:**
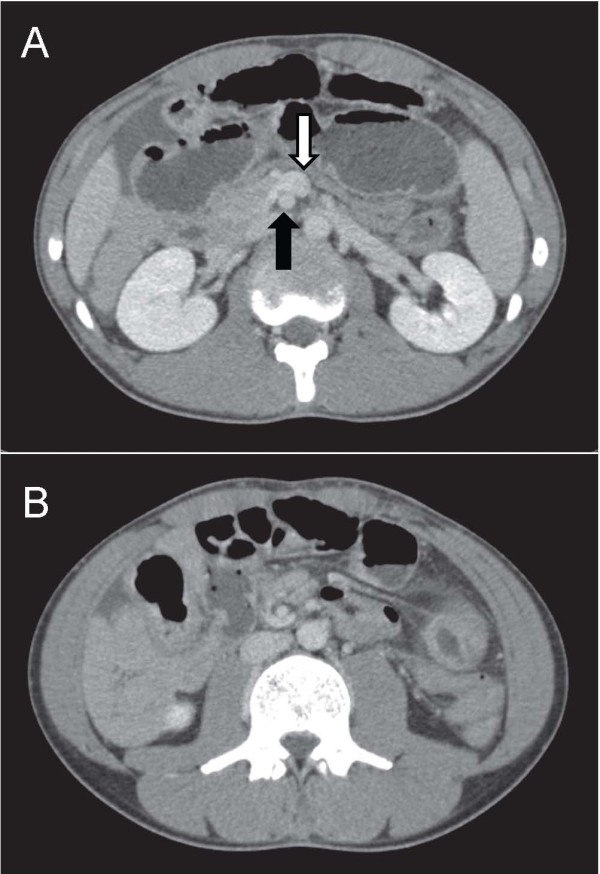
**Contrast enhanced CT of the abdomen. A**: Contrast enhanced CT can show the abnormal anatomic location of a right sided small bowel, a left-sided colon, and an abnormal relationship of the superior mesenteric vein (white arrow) situated to the anterior of the superior mesenteric artery (black arrow) instead of to the right. **B**: Opacified small bowel present almost entirely on the right side.

**Figure 2:**
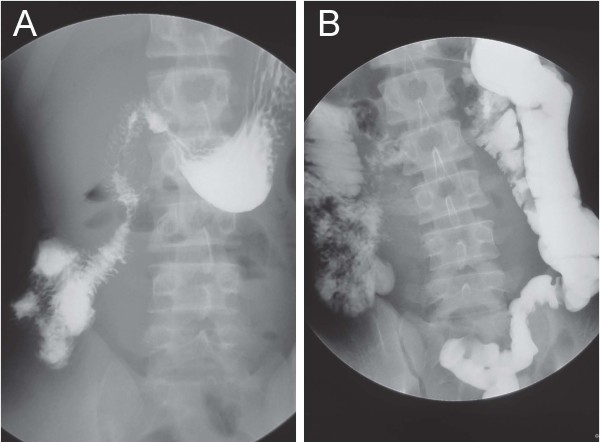
**Gastrointestinal contrast studies. A**: Upper gastrointestinal contrast studies showed malrotation of the small bowel without evidence of the duodenum crossing the lumbar spine. **B**: All small bowel was noted to be sequestered on the right side of the abdomen. The cecum lay on the left side of the abdomen and the ileum entered it from the right.

Chest radiography did not reveal any signs of perforation of a hollow viscus. Ultrasonography demonstrated a fluid-filled, distended, small gut loop. No free liquid was visible between the intestinal segments or in the pelvis. Axial contrast-enhanced computed tomography (CT) obtained through the mid-abdomen showed an inverted relationship between the superior mesenteric artery (SMA) and superior mesenteric vein (SMV). The SMV was positioned to the anterior of the SMA (Figure 1A). Opacified small bowel presented almost entirely on the right side (Figure 1B). Upper gastrointestinal tract barium studies revealed that the duodenum ran caudally in a straight line from the first part onwards. The fourth duodenal segment and the normal duodeno-jejunal junction (Treitz ligament) were not developed (Figure 2A). Barium enema revealed that all colon segments with the cecum were found to the left of the spine. The cecum lay on the left side of the abdomen and the ileum entered it from the right (Figure 2B).

Based on the diagnosis of malrotation, the patient consented to exploratory laparoscopy. No segmented gangrene of the small intestine was present. Adhesions surrounding the SMA and cecal bands attaching the duodenum and right colon were noted. The Ladd’s procedure was performed. In detail, the cecum and right colon were rotated medially to expose the duodenum. The base of the mesentery was widened by incising the peritoneum. Then, the duodenum was moved until it was oriented inferiorly toward the right lower quadrant. The entire length of bowel was examined to assure that no other obstructive bands or kinks were present. The small bowel was then placed on the right side of the abdomen, and the colon was placed on the left side of the abdomen. Finally, the appendix was removed.

Operative time was 195 minutes with negligible bleeding. Postoperative course was uneventful. The patient was discharged two days later and has remained asymptomatic without recurrence of abdominal pain three months postoperatively.

## Discussion

Malrotation of the intestinal tract is a congenital anomaly referring to either lack of or incomplete rotation of the fetal intestines around the axis of the superior mesenteric artery during fetal development. The malrotaion of the gut and abnormal location of the cecum produces a narrow superior mesenteric vascular pedicle, as opposed to the normally broadbased small bowel mesentery. This narrow superior mesenteric artery takeoff and lack of posterior peritoneal fusion predispose the patient to subsequent midgut volvulus and obstruction with potential vascular catastrophe.

Approximately 85% of malrotation cases present in the first two weeks of life [5,6]. However, presentation of intestinal malrotation is very rare and its incidence has been reported to be between 0.2% and 0.5% [7]. True incidence of malrotation in older children or adults is unclear, because a number of patients may be asymptomatic. Not all patients with malrotation present with symptoms. Even once the anomaly is discovered, many live without complaint.

In adults or older children, the difficulty of diagnosis results from both the absence of specific physical findings and the low frequency in adults [8,9]. Midgut malrotation in adults presents in numerous ways and the symptoms are non-specific. There are no typical sets of symptoms that are diagnostic of clinical problems. Symptoms in the adult patient are often mistaken for irritable bowel syndrome, peptic ulcer disease, biliary and pancreatic disease, and psychiatric disorders [8]. The location of the pain may vary from the epigastric region to the left upper abdominal quadrant, and the pain may be described as either intermittent cramping or persistent aching. It most often occurs postprandially and may last several minutes to an hour. Our patient had experienced abdominal distension, nausea, vomiting, and vague abdominal pain several times before, but the symptoms had always disappeared spontaneously.

Frequently, the plain radiograph is normal or may show an incomplete bowel obstruction. Specific findings that are diagnostic of malrotation can be detected through the use of both upper and lower gastrointestinal tract barium studies, angiography of the superior mesenteric artery, CT scan, and often emergency laparotomy. Occasionally, an abdominal radiograph will show dilated bowel loops with the orientation of a spiral nebula in the midabdomen. Barium studies may reveal a dilated duodenal loop caused by bowel obstruction with a spiral configuration of the proximal jejunal loops. CT is also used to investigate small-bowel volvulus and various signs have been described. Characteristic findings include the positioning of the superior mesenteric vein lying to the left or anterior to the artery because of torsion of the mesentery around its attachment, the presence of a right-sided duodeno-jejunal junction, the absence of a cecal gas shadow on the patient’s right side, or third and fourth duodenal junction that does not cross the patient’s spine [10,11].

Management of intestinal rotation without midgut volvulus is controversial. In general, symptomatic patients with malrotation should be treated with surgical intervention. The classic treatment for incomplete intestinal rotation is the Ladd procedure, which requires mobilization of the right colon and cecum by division of Ladd bands, mobilization of the duodenum, division of adhesions around the superior mesenteric artery to broaden the mesenteric base, and an appendectomy [12-14]. Spigland *et al.* recommended that all patients with malrotation are candidates for laparotomy, even if they are asymptomatic [15]. Mozziotti *et al.* recently reported a series of malrotation patients managed successfully with laparoscopic intervention [16]. Laparoscopy can be used to determine the position of the Treitz ligament and whether the cecum is fixed in the right lower quadrant. If the patient is decided to be at risk for volvulus (i.e. a shortened mesenteric pedicle), a Ladd's procedure can be accomplished laparoscopically with good long-term results [16,17]. Due to the abnormal cecal position inflicted by malrotation, patients with associated appendicitis will demonstrate atypical symptoms with pain projected to the left of the middle line since the appendix will not be located in the normal area in the abdomen. This could lead to confusion and delay in diagnosing appendicitis in the future. Therefore, appendectomy is usually performed during surgical intervention.

Although most of the literature consists of occasional case reports or small case series, we searched for literature published between 1983 and 2012 using PubMed and Web Japan Medical Abstracts Society and found 37 reported cases of teenage patients (ages 13 through 19) with intestinal malrotation (Table 1). Twenty patients were male and seventeen were female. The diagnosis could be made by radiographic studies in all these patients. Patients presented with a variety of gastrointestinal disorders. Abdominal pain was the most frequent symptom (30/37). Other symptoms were nausea, feeding intolerance, reflux, and respiratory problems. The Ladd procedure was performed on 27 patients; on 12 patients the procedure was conducted laparoscopically.

**Table 1:** Reported cases of intestinal malrotaion (13–19 years old)

**Year**	**Author**	**Journal**	**Age**	**Gender**	**Symptoms**	**Surgery**
**1991**	Ko, et al.	Jpn J Surg (in Japanese)	19	F	abdominal distention	Ladd procedure
**1992**	Lal, et al.	Indian J Gastroenterol	17	F	abdominal pain, vomiting	gastrojejunostomy, vagotomy
**1994**	Pelucio, et al.	Am J Emerg Med	15	M	abdominal pain	Ladd procedure
**1997**	Kimura, et al.	Jpn J Clin Surg (in Japanese)	16	M	vomiting	Ladd procedure
**1997**	Ishida, et al.	J Jpn Soc Pediatr Surg (in Japanese)	13	F	abdominal pain, vomiting	Ladd procedure
**1997**	Yahata, et al.	Surg Laparosc Endosc	17	F	abdominal pain	laparoscopic Ladd procedure
**1998**	Yokota, et al.	Kesennuma Hosp Medical J (in Japanese)	15	F	abdominal pain	Ladd procedure
**1999**	Kang, et al.	J Jpn Soc Pediatr Surg (in Japanese)	16	M	abdominal pain, vomiting	Ladd procedure
**1999**	Yamashita, et al.	Surg Endosc	13	F	vomiting	laparoscopic Ladd procedure
**2000**	Walsh, et al.	J Pediatr Surg	13	F	abdominal pain	laparoscopic Ladd procedure
**2001**	Horiba, et al.	J Jpn Clin Surg (in Japanese)	17	M	vomiting	Ladd procedure
**2003**	Tsumura, et al.	Surg Endosc	15	F	abdominal pain	laparoscopic Ladd procedure
**2003**	Singer, et al.	J Am Coll Surg	19	M	abdominal pain, vomiting	Ladd procedure
**2004**	Tseng, et al.	JBR-BTR	14	F	abdominal pain	Ladd procedure
**2005**	Sato, et al.	Hokkaido Surg J (in Japanese)	18	M	abdominal pain	release of ileus
**2005**	Kamiyama, et al.	Radiat Med	14	M	abdominal pain	Ladd procedure
**2007**	Vechvitvarakul, et al.	J Pediatr Surg	13	M	abdominal pain, nausea, vomiting	Ladd procedure, appendectomy
**2007**	Kusuda, et al.	J Abdominal Emergency Medicine (in Japanese)	17	M	abdominal pain	Ladd procedure
**2007**	Draus, et al.	Am Surg	17	F	abdominal pain, nausea	laparoscopic Ladd procedure
**2008**	Duran, et al.	Turk J Gastroenterol	17	F	abdominal pain	division of adhesions
**2008**	Uchida, et al.	J Pediatr Surg	13	F	vomiting	Bypass
**2009**	Fukushima, et al.	Jpn J Endosc Surg (in Japanese)	15	F	abdominal pain, distention	laparoscopic Ladd procedure
**2009**	Tazaki, et al.	J Abdominal Emergency Medicine (in Japanese)	14	M	abdominal pain, vomiting	release of ileus
**2009**	Shimodaira, et al.	J of Jpn Soc Psychosomatic Med (in Japanese)	17	M	vomiting	laparoscopic Ladd procedure
**2009**	Fujii, et al.	J Jpn Clin Surg (in Japanese)	14	M	vomiting	Ladd procedure
**2009**	Mano, et al.	J Jpn Soc Pediatr Surg (in Japanese)	18	M	abdominal pain	laparoscopic Ladd procedure
**2010**	Watanabe, et al.	J Jpn Soc Gastrointestinal Dis (in Japanese)	19	F	abdominal pain	release of ileus
**2010**	Takazawa, et al.	Jpn J Pediatr Surg Nutr (in Japanese)	14	M	vomiting, distention	resection of necrotic intestine
**2011**	Kokado, et al.	J Jpn Soc Pediatr Surg (in Japanese)	13	F	abdominal pain, vomiting	fixation of colon
**2011**	Lam, et al.	J Pediatr Surg	14	M	abdominal pain, vomiting	resection of necrotic intestine
**2012**	Nath, et al.	Ann R Coll Engl	16	M	abdominal pain	laparoscopic Ladd procedure
**2012**	Jain, et al.	Case Rep Radiol	15	M	abdominal pain	Ladd procedure
**2012**	Wanjari, et al.	N Am J Med Sci	17	M	abdominal pain, vomiting	laparoscopic Ladd procedure
**2012**	Macedo, et al.	Einstein	13	F	abdominal pain	laparoscopic Ladd procedure
**2012**	Tran, et al.	J Pediatr Surg	18	M	abdominal pain	Ladd procedure
**2012**	Katsura, et al.	J Jpn Clin Surg (in Japanese)	19	F	abdominal pain	resection of necrotic intestine
**2013**	Nakajima, et al.	present case	17	M	abdominal pain, vomiting	laparoscopic Ladd procedure

An important point is that since many patients with intestinal malrotation are asymptomatic, everyone in the medical community should be made aware of the problem. Also, patients with acute volvulus should be treated promptly. Some asymptomatic adults may not need surgery. Of note, there is always the possibility that laparoscopic surgery will not entirely rule out the chance of acute volvulus; it could introduce problems such as band adhesion and future adhesive small bowel obstruction.

In conclusion, a number of teenage patients with intestinal malrotation present with symptoms. Increased awareness of this condition and an understanding of its varied presentation at different ages may reduce the time needed to diagnose the problem and improve patient outcome. Laparoscopy is an excellent technique for the evaluation and definitive management of patients without midgut volvulus with intestinal rotation abnormalities.

## Consent

Written informed consent was obtained from the patient’s guardian/parent/next in keen for publication of this report and any accompanying images. A copy of the written consent is available for review by the Editor-in-Chief of this journal.

## Competing interests

The authors declare that they have no competing interests.

## Authors’ contribution

YN, HS, NY, TY, TO and MT were involved in preoperative diagnosis and postoperative care. NM conceived performed the literature search. TY, RS, SN, TS and HO performed the operation, involved in the preoperative and postoperative care. AN and JK conceived the write up, performed the literature search and drafted the manuscript. All authors read and approved the manuscript for submission.
